# Retraction: Long noncoding RNA TUG1 is a diagnostic factor in lung adenocarcinoma and suppresses apoptosis via epigenetic silencing of BAX

**DOI:** 10.18632/oncotarget.28462

**Published:** 2024-02-05

**Authors:** Huan Liu, Guizhi Zhou, Xin Fu, Haiyan Cui, Guangrui Pu, Yao Xiao, Wei Sun, Xinhua Dong, Libin Zhang, Sijia Cao, Guiqin Li, Xiaowei Wu, Xu Yang

**Affiliations:** ^1^Health Physical Examination Department of The Third Department, The First Affiliated hospital of Dalian Medical University, Dalian, Liaoning, China

**This article has been retracted:** This article contains duplicated images found in previously published papers. Specifically, Figures 4F and 5C contain images from Figure 5C and 4E of paper [1] and Figure 5C from Figure 6B of paper [2]. Therefore, with the agreement of all authors, Oncotarget has decided to retract this paper.

Original article: Oncotarget. 2017; 8:101899–101910. 101899-101910. https://doi.org/10.18632/oncotarget.22058
